# Tetanus– a case report highlighting the challenges in diagnosis and treatment

**DOI:** 10.1186/s40794-024-00220-5

**Published:** 2024-06-01

**Authors:** Menno Boer, Martijn de Voogd, Nicolasine Diana Niemeijer, Lonneke van Hoeven

**Affiliations:** Department of Internal Medicine, IJsselland Hospital, Capelle aan den IJssel, The Netherlands

**Keywords:** Tetanus, *clostridium tetani*, Vaccination, Treatment

## Abstract

Tetanus has become an increasingly rare infectious disease due to the development of successful vaccination programs in the mid-20th century. In resource-rich countries, mainly unvaccinated or partly vaccinated risk groups are affected, whereas tetanus still remains prevalent in resource-limited countries. The decreasing incidence in developed countries has hindered clinical trials evaluating the best treatment modalities for tetanus infections. Current guidelines are based on a small number of studies and case reports. So far, these studies have shown potential benefits of treating tetanus infections with benzodiazepines, magnesium sulfate and baclofen. Additionally, several treatments have been shown to be useful in stabilizing and supporting patients with tetanus. However, each treatment modality has limitations, from negative side effects to logistical challenges, especially in developing countries. Therefore, further knowledge is required to evaluate the best use of each treatment and to further optimize patient care. This knowledge can contribute to the reduction of the burden of disease in countries where tetanus remains prevalent and where resources are limited, though vaccination is the most effective method to achieve this. This case report describes the treatment of a Dutch patient with tetanus infection and illustrates the role of benzodiazepines as well as other key aspects of treating patients with tetanus.

## Background

Tetanus is a potentially deadly infectious disease, caused by toxins produced by specific strains of *Clostridium tetani* (*C. tetani*) bacteria. Spores produced by *C. tetani* can enter the body through contaminated wounds and infections can occur at any age. Patients infected with *C. tetani* can develop a range of symptoms, mostly due to loss of inhibition of motor neurons, leading to hypertonia and painful muscle spasms. If left untreated, hypertonia and autonomic dysregulation can occur, leading to changes in blood pressure, cardiac arrythmias, asphyxia and death. Its high mortality rate and the abundant presence of tetanus spores worldwide contribute to the fact that in many countries, tetanus is still a major public health issue. Currently, the majority of globally reported tetanus cases concern newborn babies and mothers who have not been sufficiently vaccinated using tetanus-toxoid-containing vaccines (TTCV) [1]. The World Health Organization (WHO) has therefore launched the Maternal and Neonatal Tetanus Elimination (MNTE) Initiative. The current focus of this project is to achieve MNTE in the 11 remaining countries that currently do not meet these standards. Thereafter, WHO efforts will be focused on maintaining elimination of maternal and neonatal tetanus [2]. 

During the early to mid-20th century, tetanus was a serious global concern due to its high incidence and mortality, with an estimated case-fatality rate of nearly 100% in the absence of medical intervention [1]. However, tetanus has become increasingly rare ever since the introduction of vaccinations against tetanus toxins in the mid-20th century [3]. The incidence of tetanus cases is still steadily declining since the first development of TTCV in 1926. It has been shown that the global incidence of tetanus cases has decreased by 88% between 1990 and 2019, nevertheless, a total number of 73,662 cases were reported in 2019 worldwide [4]. Recent epidemiological data show a total of 50 reported cases in 2021 in 26 countries belonging to the European Union (EU) or European Economic Area (EEA) [5]. Only 10 of the aforementioned cases have been classified as confirmed, with 40 out of 50 cases classified as probable [5]. 

This decline in incidence has mostly depended on increasing TTCV vaccination rates, such as in Western Europe. Areas with higher TTCV vaccination rates have reported remarkably fewer tetanus cases than areas in which TTCV vaccination rates are lower [4–6]. Since the introduction of diphtheria, tetanus toxoid, and pertussis vaccines (DTP3, a form of TTCV) in childhood vaccination programs, vaccination rates in Western Europa quickly reached 80%, as recommended by the WHO [6]. For the past decades, vaccination rates in Western Europe have even been as high as 95%, as shown in Table 1, in contrary to countries in Sub-Saharan Africa, such as Somalia, where vaccination rates have yet to exceed 50% [6]. 

**Table 1:** DTP3 vaccination rates as percentages of one-year-olds immunized in North-Western Europe [6]

Country/area	1980	2021	Absolute Change	Relative Change
Belgium	95%	98%	+ 3 pp	+ 3%
Denmark	88%	97%	+ 9 pp	+ 10%
Finland	92%	89%	-3 pp	-3%
Germany		91%		
Luxembourg		99%		
Netherlands	96%	95%	-1 pp	-1%
Norway		97%		
Sweden	99%	98%	-1 pp	-1%
United Kingdom*	41%	93%	+ 52 pp	+ 127%
Other				
World	20%	81%	+ 61 pp	+ 305%

Abbreviations: pp, percentage point

*The DTP3 vaccination rates in the United Kingdom in 1980 were significantly lower as a result of the “pertussis crisis”. Public debate regarding the safety of DTP3 vaccines in the mid-1970s caused the vaccination rates to drop to below 40%. DTP3 vaccination rates recovered in the mid-1980s [44]

**Fig. 1:**
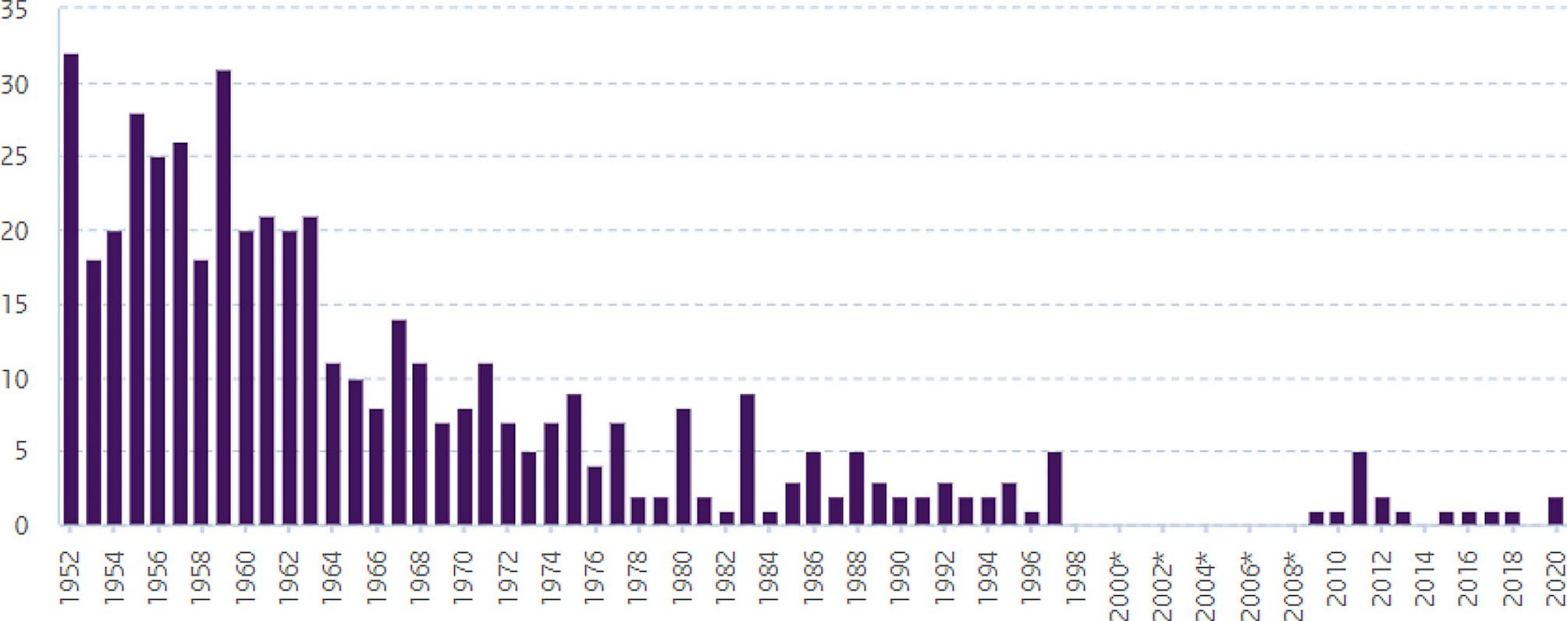
Number of tetanus cases in The Netherlands from 1952 to 2020* [7]. *Tetanus was not a notifiable disease between 1998 and 2008

The Dutch Governmental institute of Healthcare and Environment (Rijksinstituut Volksgezondheid en Milieu, RIVM) have reported a total number of 478 tetanus cases since 1952, with 308 reported deaths [7]. Since the introduction of TTCV in the national childhood vaccination program in 1957, the incidence has drastically decreased from 26 annual cases (1957) to 2 cases (2020), a 92% decline, as shown in Fig. 1. Only 5 deaths following a tetanus infection have been reported since 2000, the last of which was reported in 2011 [7]. Despite the successful vaccination program against tetanus infections, 0 to 5 annual cases are reported in the Netherlands, mostly concerning unvaccinated or partly vaccinated risk groups [7]. Such risk groups include patients older than 65 years, patients with diabetes mellitus or a history of immunosuppression, and intravenous drug users. Immigrants with an unclear vaccination status may also be at risk [1, 8]. 

While both incidence and prevalence decline, challenges arise in developing treatment options for those who have been infected by *C. tetani*. Due to the low incidence, research towards finding adequate treatment is challenging. However, the mortality of tetanus worldwide is still substantial, hence both experience and knowledge on the efficacy of various treatment options are essential. Therefore, we present a case of a patient infected with *C. tetani*, admitted to and treated in the Internal Medicine Clinical Ward of the IJsselland Hospital in the Netherlands.

## Main text

An eighty-year-old woman was admitted to the Emergency Department (ED) due to dehydration. Her medical history included a recently diagnosed polymyalgia rheumatica, currently treated with 30 milligrams of oral prednisolone daily. Complaints of a trismus had developed four days prior to her presentation and had made oral intake impossible. The patient had already consulted her own rheumatologist, an orofacial physical therapist, a maxillofacial surgeon and a neurologist, none of whom could find the cause of her complaints. In addition to the trismus, the patient presented with evident generalized hypertonia and remarkable diaphoresis. Patient history was negative for fever, recent traumas or animal bites.

Physical examination showed a slightly elevated respiratory rate (20 breaths per minute), a mild tachycardia (95 beats per minute) with a non-invasively measured blood pressure of 157/87 mmHg. Her body temperature was 36.7 °C, and peripheral pulse oximetry saturation was normal (95%).

Laboratory results showed a mild leukocytosis (15.3 × 10^9^/L) with an increased neutrophil count (12.8 × 10^9^/L), and slightly elevated C-reactive protein levels (22 mg/L). Additional laboratory results, including kidney function, liver function, electrolyte levels, and creatin kinase levels, showed values within normal range. The patient was admitted to the department of Internal Medicine for further clinical observation. On the first day after admission, a more extensive patient history revealed that the patient had suffered from a fall in her backyard three weeks prior to ED admission. The fall had resulted in a wound on her lower leg, after which she had visited her general practitioner, who sutured the wound and administered a TTCV catch-up vaccination. Based on this new information, blood- and wound cultures were obtained and treatment with intravenous antibiotics (amoxicillin and clavulanic acid) as well as high-dosed tetanus immune globulin was started. Nevertheless, complaints of hypertonia and painful muscle spasms persisted.

To relieve the complaints of muscle spasms and rigidity, the patient was started on intravenous benzodiazepines (diazepam, 2.5 milligrams three times daily). While the effect of this treatment was evaluated and appeared to provide little relief, the wound cultures came back positive for *C. tetani*. Due to the presence of three other bacteria in the wound cultures (Escherichia coli, Pseudomonas aeruginosa, and Enterobacter cloacae), antibiotic treatment was switched to broad spectrum antibiotics (meropenem, 1000 milligrams three times daily) for a period of two weeks. In conformity with current treatment guidelines, surgical wound debridement was performed, a second round of high-dosed tetanus immune globulin was administered, and a full TTCV vaccination regimen was administered. Diazepam dosages were gradually increased, eventually to a total dosage of 40 milligrams daily, with moderate effect on muscle spasms and trismus. Treatment was continued for two weeks; during this period inflammatory markers normalized and the wound showed adequate healing.

One month after being admitted to the Internal Medicine department, the patient had recovered enough to be discharged for further rehabilitation. Diazepam dosages were tapered to 5 milligrams three times daily. The patient received her second dose of TTCV before being discharged. Six weeks after being discharged, the patient visited the outpatient clinic and had nearly fully recovered. Due to the use of prednisolone during the vaccination regimen, tetanus immunoglobulin levels will be analyzed one month after the last TTCV vaccination to evaluate the immune response. Anti-tetanus toxoid IgG levels greater than 0.15 IU/ml will be considered as adequate protection [9]. 

## Discussion

**Fig. 2:**
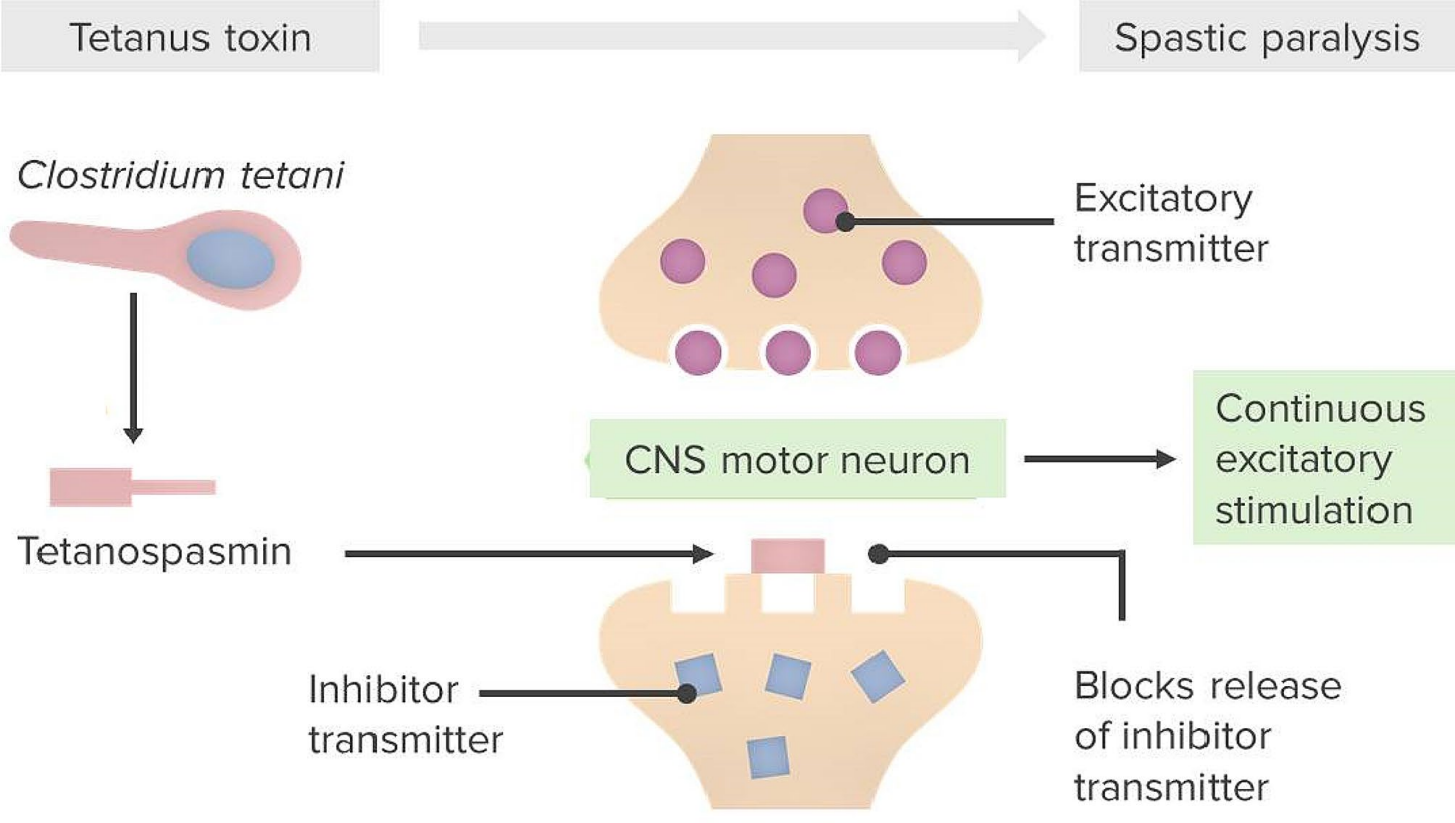
Illustration of the mechanism of action of tetanus toxin [45]. schematic illustration of pathophysiology in tetanus induced spastic paralysis: in an anaerobic environment (e.g. during active inflammation from the contaminated tissue) *C. tetani* spores germinate and produce the tetanospasmin toxin. Tetanospasmin binds to the presynaptic neuron, eventually allowing the light chain part of the protein to reach the spinal cord. The toxin specifically enters the central inhibitory neurons, prohibiting the release of GABA- and glycine containing vesicles from the cell membrane. This results in a loss of inhibition and subsequently continuous excitatory stimulation in motor neurons and the autonomic nervous system, causing uncontrolled motoric contractions [46]

Studies have elucidated the pathophysiological process of tetanus infections, which starts with the production of metalloprotease tetanus toxin, otherwise known as *tetanospasmin* [1, 3]. Tetanus toxin is produced by *C. tetani*, after spores of *C. tetani* have inoculated infected human tissue. The tetanus toxin is subsequently transported to the peripheral nervous system through blood and lymphatic vessels. Retrograde axonal transport then allows the tetanus toxin to reach the central nervous system, where it enters inhibitory interneurons. Inhibitory interneurons affected by tetanus toxins lose their ability to inhibit anterior horn cells and autonomic neurons, resulting in hypertonia, muscle spasms and autonomic dysregulation [3]. This process has been illustrated in Fig. 2.

Treating tetanus starts with adequate antibiotic therapy, wound cleaning and neutralization of circulating antibodies using tetanus immune globulins [1, 3, 10]. The importance of adequate wound cleaning has been illustrated in two cases where *C. tetani* was still found in wound cultures despite 16 days of intravenous penicillin [11]. Equine tetanus immune globulins were first developed in 1910, with humane tetanus immune globulins (HTIG) becoming available in the 1960s [12]. From that moment on the use of HTIG has become standard practice in the treatment of tetanus in resource-rich countries, since equine immune globulins may induce allergic reactions [1, 10, 13]. However, HTIG are still costly and may be difficult to acquire in resource-limited countries. In such countries, equine tetanus immune globulins are more commonly used, despite its risk of concomitant anaphylaxis [13]. The ideal use of HTIG is still a subject of research, with current studies mainly focusing on the potential benefits of intrathecal administration of HTIG in comparison to intravenous or intramuscular administration. So far, several studies have compared the effects of different routes of administration and have shown potential benefits of intrathecal HTIG [14–16]. Such benefits include a significant reduction in mortality, hospital stay, and an improvement in controlling muscle spasms [14–16]. However, subsequent meta-analyses have provided conflicting results, leaving the optimal route of administration a subject to discussion [17, 18]. 

Though the clinical advantages of antibiotic therapy in patients with tetanus have not yet been established, antibiotic therapy should always be considered due to possible coinfection by other bacteria. Evidence supporting the role of antibiotic treatment in tetanus is, however, scarce. One of the earliest studies was conducted in 1985, and favored metronidazole over penicillin [19]. These results were contradicted when a study compared benzathine penicillin, benzyl penicillin and oral metronidazole, and found no significant difference in hospital stay, use of neuromuscular blockade or the need for mechanical ventilation [20]. Currently, metronidazole and penicillin G are the preferred drugs of choice regarding tetanus infections [1, 10, 21]. 

During both treatment and the recovery period, managing symptoms still poses a challenge. Key aspects of alleviation of symptoms include the reduction of muscle rigidity, muscle spasms and autonomic dysregulation [3, 10]. Current literature is scarce, however increased survival rates have been achieved with the use of sedation and muscle relaxants, combined with mechanical ventilation if necessary [10]. A suitable option to achieve muscle relaxation is the use of benzodiazepines, such as diazepam and midazolam (enhancement of the binding of gamma-aminobutyric acid (GABA) to its receptor) [10, 21, 22]. However, high quality evidence on the use of benzodiazepines and their optimal utilization is lacking, in part due to ethical limitations to studies required to provide such evidence.

An alternative treatment consists of the use of intravenous magnesium sulfate. Its value in controlling muscle spasms secondary to tetanus infections was first established in the 1980s [23]. Since then, more evidence has supported the beneficial effects of magnesium sulfate. However, only a small number of randomized clinical studies have been performed comparing magnesium to placebo. The studies that have compared magnesium to placebo have not provided conclusive evidence that magnesium decreases the need for mechanical ventilation [24]. A meta-analysis of three studies concerning treatment using magnesium showed that magnesium did not reduce overall mortality in tetanus, though these studies have shown beneficial effects in controlling muscle spasms and autonomic dysregulation [25]. 

Finally, baclofen (a derivative of gamma-aminobutyric acid) can be considered for the treatment of severe muscle spasms. Oral baclofen is, however, deemed ineffective due to its poor penetration across the blood-brain-barrier. Therefore, baclofen should be administered intrathecally, which is expensive and limited to specialized clinics. Its potential benefits have only been described in case reports and high-quality evidence comparing intrathecal baclofen to other modes of treatment is lacking [26–36]. Moreover, these case reports have reported adverse effects such as hemodynamic instability and the need of ventilatory support secondary to respiratory depression. Consequently, use of intrathecal baclofen is currently not recommended according to the current literature and little is known about its ideal application in the alleviation of muscle spasms [10]. 

While treatment of muscle spasms is necessary in almost all cases of tetanus, some cases require additional treatment of autonomic dysregulation. Several treatment modalities have been reported to be effective, though their evidence consists of only a small number of case reports [10]. One of the first drugs that was used to treat autonomic dysregulation in tetanus patients, specifically tachycardia and hypertension, was labetalol (a non-selective ß-adrenergic receptor antagonist) [37]. Labetalol has been shown to be useful in cases of adrenergic crises and can reduce subsequent tachycardia and hypertension [3, 22, 38, 39]. However, labetalol does not reduce variability in heart rate and blood pressure, and in some cases co-administration of clonidine (α_2_-adrenergic receptor agonist) is necessary to achieve adequate response. Treatment with intravenous clonidine alone has also been studied and has been reported to be effective in reducing blood pressure fluctuations and mortality [40]. In cases where adrenergic blockers are unavailable or otherwise unfavorable, intravenous morphine can be used. Intravenous morphine, partially due to its analgesic effects, has been shown to successfully control autonomic dysregulation [41]. 

In addition to treatment of muscle spasms and autonomic dysregulation, all tetanus patients should receive supportive care as needed [3, 21, 22]. Due to high metabolism and energy demand, suppletion of fluids and parental feeding should be considered in patients whose oral intake is diminished due to trismus [3]. 

In resource-limited areas, the aforementioned therapies may not always be readily available. In order to reduce the incidence of maternal and neonatal tetanus, adequate preventative measures are advised by the WHO. Such measures include, but are not limited to, the vaccination of women of childbearing age, use of sterile instruments during deliveries, disinfection of surfaces and protection of the umbilical stump to prevent infection. Since tetanus is a disease with high morbidity and significant mortality regardless of gender or age, intensification of tetanus vaccination programmes for general populations is necessary to further reduce and ultimately minimalize the incidence in resource-limited countries. If infection does occur, use of equine tetanus immune globulin may be considered in order to prevent worsening of symptoms. Symptoms may be managed by high dosed diazepam, or continuous midazolam infusions, since magnesium sulphate and intrathecal baclofen are likely unavailable [5, 13, 21]. 

Prevention of tetanus infections by increasing vaccination rates and adequate post-exposure prophylaxis remains key in decreasing the incidence of tetanus infections worldwide. Post-exposure prophylaxis using TTCV and HTIG depends on wound characteristics and whether patients have previously been vaccinated using TTCV or not. Current literature recommends catch-up vaccination using TTCV in patients with clean, minor wounds and an unknown TTCV-vaccination status or patients who have received less than three previous doses of TTCV in their lifetime. Use of HTIG is not indicated in patients with clean, minor wounds. Additionally, if patients have three or more previous TTCV-vaccinations but the last dose was given more than ten years ago, a catch-up vaccination using TTCV is advised. Patients who suffer from larger or unclean wounds should receive TTCV if they have not been previously vaccinated using TTCV, or if they have previously received less than three doses of TTCV. Catch-up vaccination using TTCV is advised if the last dose was given more than five years ago. Use of HTIG is only recommended in patients with larger, contaminated wounds who have received less than three TTCV-vaccinations or if their vaccination status is not known [42]. 

Dutch national guidelines regarding post-exposure prophylaxis recommend similar uses of TTCV-vaccines and HTIG. These guidelines do not discriminate between size and or contamination of wounds, but recommend post-exposure prophylaxis in patients presenting with (possibly) contaminated open wounds, wounds resulting from animal bites and second- or third-degree burns. Patients with a full TTCV vaccination history who have received their last dosage of TTCV-vaccines less than ten years ago do not require additional vaccination. For male patients born after 1936 and female patients born after 1950 with a presumed full vaccination history, it is recommended to administer TTCV-catch up vaccination. This is based on conscription of male patients in military service, requiring vaccination against tetanus due to increased risk of exposure. Male patients born before 1936 or female patients born before 1950 are recommended to receive a TTCV-catch up vaccination and HTIG. Patients who have not (fully) been vaccinated with TTCV should receive both HTIG and a full TTCV vaccination regimen at 0, 1 and 7 months after exposure [43]. In retrospect, the patient presented in this case report should have received a TTCV-catch up vaccination, HTIG, and the full TTCV-vaccination regimen after her first visit to the general physician, since she was born in 1943.

## Conclusions

Tetanus is a life-threatening infection and can only be prevented by active immunization using TTCV. The success of TTCV has been illustrated by the steady decline in incidence of tetanus infections, especially in areas with high vaccination rates such as Western Europe. Patients infected by *C. tetani* can suffer greatly from hypertonia, trismus, painful muscle spasms and autonomic dysregulation. Treating these symptoms can be challenging, since evidence supporting different treatment modalities is limited. Key aspects of treatment include wound debridement, neutralization of tetanus toxin using tetanus immune globulin, active immunization, controlling muscle spasms and managing autonomic dysregulation. Muscle spasms can be reduced using benzodiazepines, magnesium sulfate and intrathecal baclofen. Autonomic dysregulation may be limited by the use of labetalol, clonidine or intravenous morphine. This case report illustrates successful treatment of a patient with tetanus and the efficacy of diazepam in controlling muscle spasms. Experiences regarding treatment of tetanus are scarce due to the declining incidence, especially in resource-rich countries. Nevertheless, worldwide tetanus is still a major public health issue; in addition to increasing vaccination rates, it remains essential to further improve patient care.

## Data Availability

No datasets were generated or analysed during the current study.

## Abbreviations

*C. tetani*: *Clostridium tetani*
DTP3: Diphtheria, tetanus toxoid and pertussis vaccines
ED: Emergency Department
EU: European Union
EEA: European Economic Area
HTIG: Human Tetanus Immune Globulins
MNTE: Maternal and Neonatal Tetanus Elimination
TTCV: Tetanus–toxoid–containing vaccines
WHO: World Health Organization
